# Neuroimaging characteristics and growth pattern on magnetic resonance imaging in a 52-year-old man presenting with pituicytoma: a case report

**DOI:** 10.1186/1752-1947-6-306

**Published:** 2012-09-18

**Authors:** Yasushi Kosuge, Jun Hiramoto, Hiroyuki Morishima, Yuichiro Tanaka, Takuo Hashimoto

**Affiliations:** 1 Department of Neurosurgery, St. Marianna University School of Medicine, 2-16-1 Sugao, Miyamae-ku, Kawasaki, Kanagawa, 216-8511, Japan

**Keywords:** Neurohypophysis, Pituicyte, Pituicytoma, Tumor volume doubling time

## Abstract

**Introduction:**

Pituicytoma is a rare neoplasm of the neurohypophysis. To the best of our knowledge there have been no reports of pituicytoma in which long-term magnetic resonance imaging observation was performed. We calculated the doubling time of the tumor volume and described the growth pattern of a pituicytoma.

**Case presentation:**

A 52-year-old Japanese man with a history of decreased libido was found to have a sellar and suprasellar mass. He underwent transsphenoidal surgery, but only a small specimen was obtained because of intraoperative bleeding. The tentative histological diagnosis was schwannoma. He noticed bitemporal hemianopsia 7 years later. A follow-up magnetic resonance imaging disclosed a tumor volume doubling time of 3830 days. Transcranial gross-total tumor resection was performed. The lesion consisted of elongated and plump tumor cells that were arranged in a fascicular or storiform pattern and were positive for S-100 protein and focally positive for glial fibrillary acidic protein. The final histological diagnosis was pituicytoma.

**Conclusion:**

Pituicytoma is a slow-growing tumor, but the growth rate may change during follow-up.

## Introduction

Pituicytoma is a rare neoplasm in adults. It corresponds to a low-grade astrocytoma of the neurohypophysis that presumably arises from pituicytes of the stalk and posterior lobe of the pituitary gland [1,2]. This tumor is included in the 2007 World Health Organization (WHO) classification of tumors of the central nervous system as a WHO grade 1 tumor [3]. We report a case of pituicytoma and discuss the neuroimaging characteristics and surgical findings.

## Case presentation

**Figure 1:**
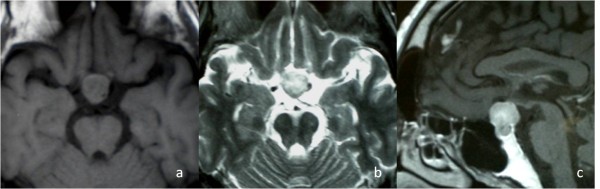
** Initial magnetic resonance imaging. A**) T1-weighted magnetic resonance image showing an isointense pituitary mass. **B**) T2-weighted magnetic resonance image showing a hyperintense mass.

A 52-year-old Japanese man presented with a 1-year history of decreased libido. He was referred to our institution for evaluation of a possible pituitary tumor. Laboratory tests were all within normal limits except for a low free testosterone level. A visual field defect in his upper right quadrant was found in a neuro-ophthalmologic examination. Magnetic resonance imaging (MRI) revealed a 20mm mass that was isointense on T1-weighted MRI and hyperintense on T2-weighted MRI. The lesion was homogeneously enhanced with gadolinium (Figure 1). Computed tomography (CT) scans showed strong contrast enhancement.

**Figure 2:**
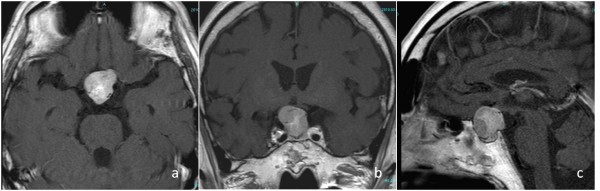
** Magnetic resonance imaging at 7 years after initial surgery. A**–**C**) Axial, coronal and sagittal contrast-enhanced magnetic resonance image showing enlargement of the tumor.

Transsphenoidal tumor removal was attempted, but only a small specimen was obtained because control of tumor bleeding was difficult. Hematoxylin-eosin staining revealed spindle cells with an eosinophilic cytoplasm. On immunohistochemical staining, the tumor cells were positive for S-100 protein, but negative for glial fibrillary acidic protein (GFAP) and cytokeratin. The patient’s molecular immunology Borstel-1 (MIB-1) proliferation index was less than 2%. The tentative histological diagnosis was schwannoma. Hydrocortisone and levothyroxine sodium hydrate were administered postoperatively. The visual field testing did not show any change from his preoperative testing. We recommended a second transcranial surgery or radiotherapy, but the patient refused both proposed treatments. Therefore, careful observation has subsequently been performed with yearly MRI. This follow-up imaging showed quite slow growth in the first 5 years after surgery, with a tumor volume doubling time (TVDT) of 20,267 days. Progressive bitemporal hemianopsia occurred at 7 years after surgery and MRI revealed apparent tumor progression during the previous 2 years (Figure 2). The TVDT during these 2 years was 1369 days and the overall TVDT over 7 years was 3830 days.

**Figure 3:**
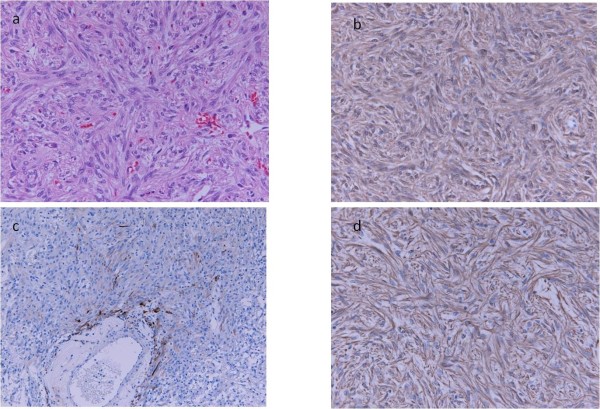
** Microscopic findings. A**) Microphotograph of an hematoxylin and eosin-stained section showing bipolar spindle cells arranged in a storiform pattern. **B**) S-100 immunohistochemical stain. **C**,**D**) Immunohistochemical stains were focally positive for glial fibrillary acidic protein and positive for vimentin.

The patient finally decided to undergo surgery to remove the tumor. A second operation was performed for transcranial tumor removal with an interhemispheric approach. The tumor was soft and hemostasis of tumor bleeding was difficult. The surgical removal was gross total. The postoperative course was uneventful except for transient diabetes insipidus. Clinical examination showed an improvement of visual field defects. There has been no recurrence for 1 year postoperatively. Histologically, elongated and plump tumor cells were arranged in a fascicular or storiform pattern and contained eosinophilic cytoplasm. Immunohistochemical stains were positive for S-100 protein and vimentin, focally positive for GFAP, and negative for synaptophysin, epithelial membrane antigen (EMA), and periodic acid-Schiff (PAS). The patient’s MIB-1 index was 1% (Figure 3). All these findings are compatible with a diagnosis of pituicytoma.

## Discussion

Pituicytoma is a very rare tumor of the adult neurohypophysis with only about 50 reported cases [4]. Several authors have also used the term ‘pituicytoma’ to refer to other tumors in the neurohypophysis, especially pilocytic astrocytoma and granular cell tumor. The 2007 WHO classification of tumors of the central nervous system defines pituicytoma as a low-grade glial neoplasm of the neurohypophysis or infundibulum that originates from pituicytes [3].

The neuroimaging characteristics of pituicytomas are nonspecific. Pituicytomas have been described to be located within the sellar, the suprasellar region or both [4]. Although there were not many reports on the CT findings of pituicytomas, in the case reported by Wolfe et al. [2], the tumor showed sellar enlargement and bony remodeling on CT scan. MRI shows an isointense mass on T1-weighted images, hyperintensity on T2-weighted images, and homogeneous enhancement with gadolinium. Therefore, the radiological differential diagnosis should include other sellar or suprasellar tumors including meningioma, craniopharyngioma, hemangiopericytoma, granular cell tumor, and pilocytic astrocytoma [4]. It is often difficult to identify these tumors in preoperative neuroradiological findings, especially for nonfunctioning pituitary adenoma in our case. However, the histologic appearance and immunohistochemical findings are distinctive [2,3]. Pituicytomas contain elongated spindle cells that are arranged in a bundle or storiform pattern with no granular component. Staining is positive for S-100, focally positive for GFAP and EMA, and negative for PAS. In the current case, we believe that the resected specimen in the first surgery stained negatively for GFAP because of the minimal resection that was possible in this surgery.

In previous reports, the most common symptoms at presentation of pituicytoma have been headache (38.9%) followed by visual field defect (31.5%). Only one patient presented with diabetes insipidus, despite pituicytomas arising from the posterior pituitary gland or infundibulum [4]. Nakasu et al. suggested that pituicytomas start to grow in the posterior lobe or lower portion of the stalk, so the upper neurohypophysis might have to compensate for the loss of function [5].

Surgery is the main treatment for pituicytoma [6]. Tumor recurrence has occurred in some patients after subtotal resection, but there have been no reports of recurrence or enlargement in patients who underwent total or gross total resection [1,5]. Thus, the best chance for cure appears to be gross total resection because partial resection carries a significant possibility of recurrence [2]. In our first surgery, we were able to achieve only a biopsy because of significant bleeding. Several other reports have also described considerable bleeding during surgery [1-3,5-14], whereas others have indicated that bleeding was easily controlled [4,5]. We compared the tumor size for these two types, based on definition of the sagittal diameter of the pons as 26.6mm [15] and estimation of the tumor size from sagittal MRI. The mean diameter did not differ significantly between hemorrhagic tumors (22.9mm) and non-hemorrhagic tumors (21.4mm). Therefore, the risk of intraoperative bleeding does not seem to depend on the tumor size. Some patients who underwent subtotal resection were also treated with postoperative radiotherapy [1,7,8]. However, the value of adjuvant therapy for pituicytomas is anecdotal and unclear in the absence of evidence from a large series [2,6].

**Figure 4:**
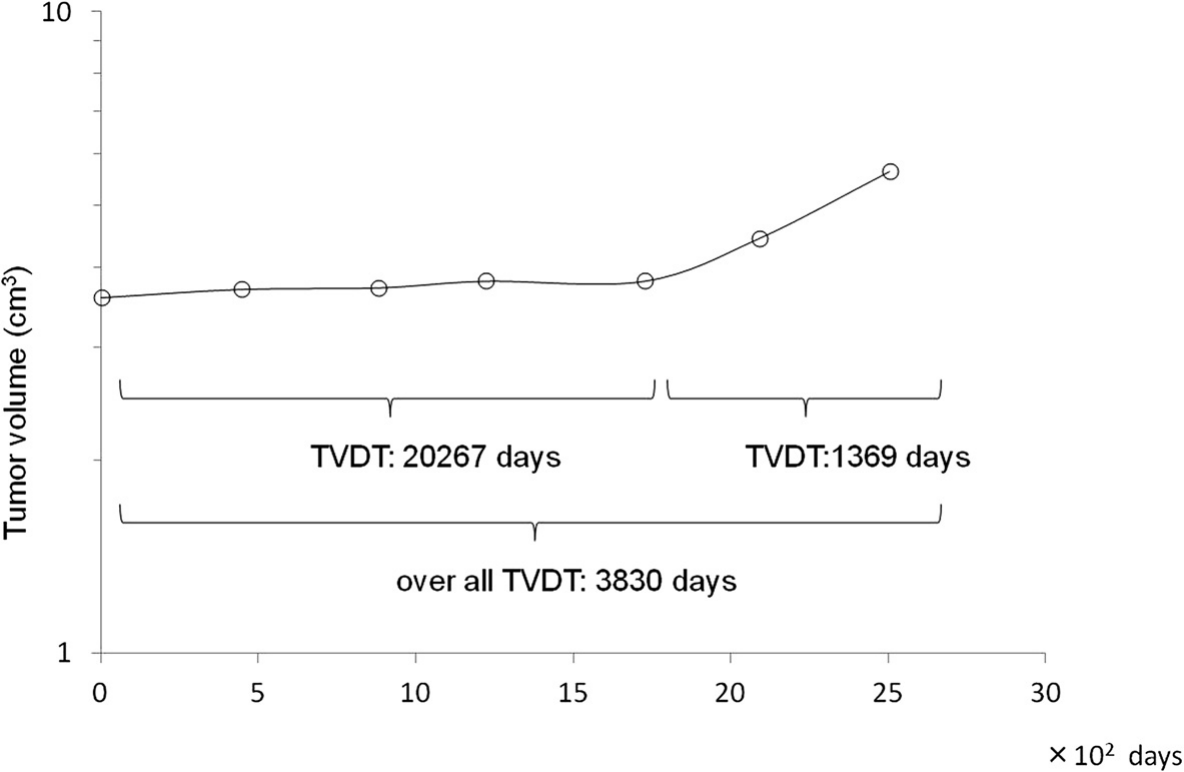
** A semi-logarithmic graph showing serial changes of the tumor volume.** The tumor volume doubling time (TVDT) was 3830 days during the follow-up period of 7 years, with TVDTs of 20267 days over the first 5 years and 1369 days over the last 2 years.

The natural history of pituicytomas is unknown [6]. We performed MRI follow-up for 81 months after biopsy and measured sequential changes in the tumor volume (Figure 4), which was computed by summing the tumor areas of all slices and multiplying by the slice thickness. This permits evaluation of the natural progression of pituicytoma, and to the best of our knowledge this is the first report of the growth pattern of a pituicytoma. The TVDT over 7 years was 3830 days, including a period of very slow growth in the first 5 years, during which the TVDT was 20,267 days. The tumor then rapidly enlarged, with a TVDT of 1369 days over the last 2 years. It is unclear why the growth rate changed during the postoperative course, but surgery might have been an influential factor. There is also a possibility that pituicytomas may enlarge rapidly without malignant change. The relatively slow growth of pituicytomas and the possible change in growth rate during follow-up should be recognized in determining the most appropriate treatment approach, although accumulation of more cases is necessary to confirm these findings.

## Conclusions

Pituicytoma is an extremely rare tumor of the neurohypophysis. To the best of our knowledge this is the first report of the TVDT of pituicytoma. Pituicytoma is a slow-growing tumor, but may enlarge rapidly without malignant change. The relatively slow growth of pituicytoma and the possible change in growth rate during follow up should be recognized in determining the most appropriate treatment approach.

## Consent

Written informed consent was obtained from the patient for publication of this case report and any accompanying images. A copy of the written consent is available for review by the Editor-in-Chief of this journal.

## Competing interests

The authors declare that they have no competing interests.

## Authors’ contributions

YK drafted and edited the manuscript. JH, HM, YT and TH contributed to the writing of the manuscript. All authors read and approved the final manuscript.
